# Thermal, spectroscopic, SEM and rheological datasets of native and quaternized guar gum

**DOI:** 10.1016/j.dib.2020.105565

**Published:** 2020-04-18

**Authors:** Rakhi Tyagi, Pradeep Sharma, Ajeet K. Lakhera, Vineet Kumar

**Affiliations:** Chemistry and Bioprospecting Division, Forest Research Institute, Dehradun-248006, India

**Keywords:** TGA/DTA, SEM, NMR, Viscosity

## Abstract

The manuscript reports TGA, DTA, SEM and NMR datasets of native and quaternized guar gum as a tool for their characterization. The TGA and DTA data was acquired in temperature range 35–500 °C. Based on the TGA experimental values, activation energy plots were drawn to study the stability of native and quaternized polysaccharides (Cai and Bi, 2008 [1]). The data demonstrates the thermal behaviour of quaternized guar gum vis-a-vis native guar gum. The surface morphology of native and quaternized galactomannan was represented by SEM imaging. The ^1^H–^1^H COSY was acquired to understand structural changes by quaternization. The rheological measurements of native and modified products were carried out to obtain the viscosity profile of the respective samples. The datasets support the research article ‘Synthesis of quaternized guar gum using Taguchi L (16) orthogonal array’ (Tyagi et al., 2020 [2]).

Specifications table

**Table:** 

Subject	Chemistry
Specific subject area	Chemical modification
Type of data	Table
	Image
	Graph
	Figure
How data were acquired	Thermogravimetric: DTG-60 unit (Shimadzu, Japan) under the argon atmosphere.
	NMR analysis: Bruker Avance Neo 500 MHz NMR spectrometer.
	Rheological data: Brookfield DV-III Ultra digital Viscometer.
	SEM imagining: SEM-Zeiss EVO-40 EP, Magnification: up to 10,00,000 X, Resolution: 30 nm (HV SE), Carl Zeiss AG Company, Germany.
Data format	Raw and analysed data
	A. TGA/DTA analysis with activation energy curves of native and quaternized guar gum
	B. SEM images of native and quaternized gum
	C. NMR spectra of native and quaternized guar gum
	D. Viscosity analysis of native gum and quaternized product
Parameters for data collection	TGA/DTA data collection was carried out under the argon atmosphere. The data was collected at a heating rate of 10 °C/min from 35 to 500 °C.
	SEM data was collected by coating the samples with gold
	COSY NMR spectrum was recorded at 125.77 MHz
	Number of scans: 4
	Relaxation delay: 0.5351 s
	Acquisition time: 0.6144s
	Temperature: 26 °C
	Solvent: D_2_O
	Data analysis was processed using iNMR software, version 5.5.5.
Description of data collection	Native guar gum and synthesised derivatives were purified and used for TGA/DTA analysis and NMR spectral recording. The electron microscope imaging and rheological analysis were also performed for these samples.
Data source location	Institution: Forest Research Institute, Dehradun
	Wadia Institute of Himalayan Geology, Dehradun
	Sophisticated Analytical Instrument Facility (SAIF), Punjab University, Chandigarh
	City: Dehradun, Chandigarh
	Country: India
Data accessibility	The raw data files are provided as supplementary files.
	All other data is with this article
Related research article	The data is related to the research article:
	Authors: Rakhi Tyagi, Pradeep Sharma, Raman Nautiyal, Ajeet K. Lakhera and Vineet Kumar. Synthesis of quaternized guar gum using Taguchi L(16) orthogonal array. Carbohydrate Polymers, Volume 237, 1 June 2020, 116136*.*
	https://doi.org/10.1016/j.carbpol.2020.116136

## Value of the data

• The datasets show thermal and rheological behaviour of optimized quaternized derivative which will provide understanding for improved utilization of modified products.
• NMR spectral data provided information about structural changes by quaternization of guar gum.
• The surface morphological changes influenced by derivatization is observed in SEM imaging.
• Researchers can use this data to understand quaternization of polysaccharides.
• The data can also be used as reference for future studies on modification of biopolymers.

## 1 Data description

**Fig. 1:**
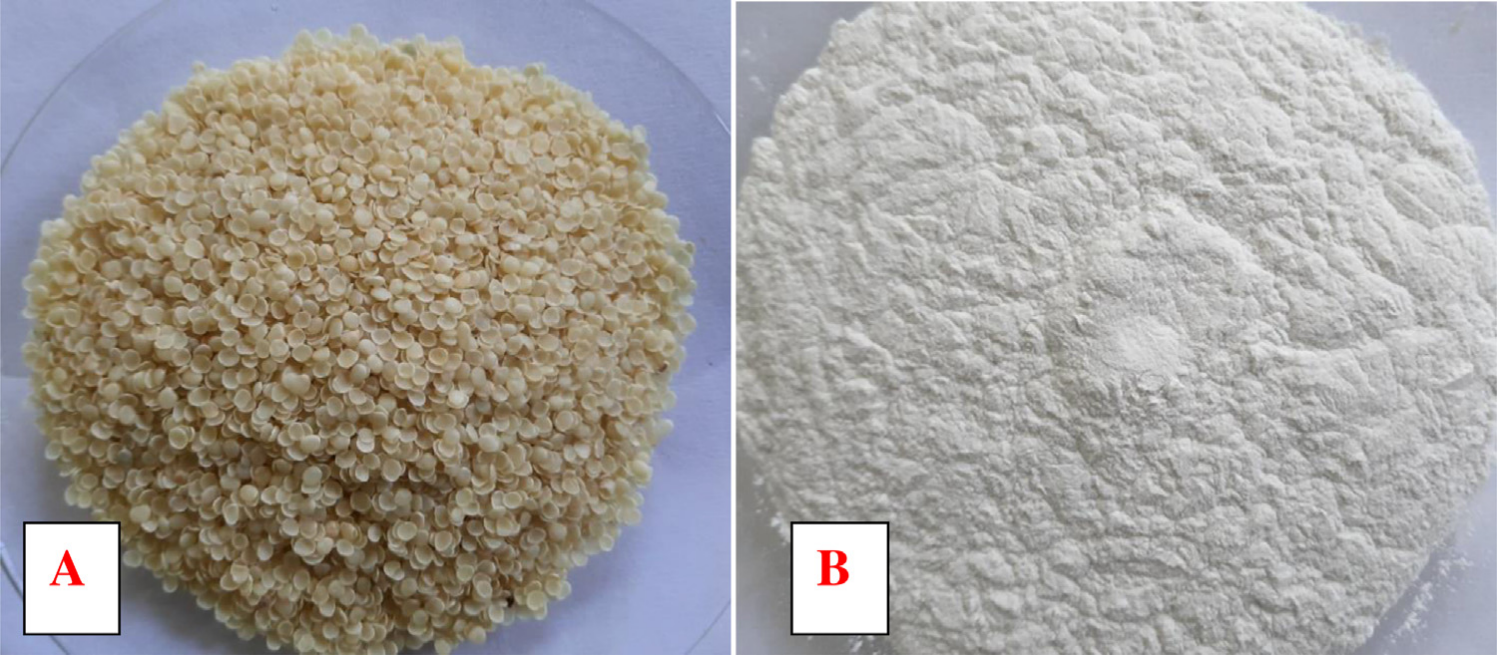
Images of Guar splits (A) and Guar gum powder (B).
• Fig. 2: Thermo-gravimetric plots: TGA of native (A) and quaternized (B) guar gum; DTA of native (C) and quaternized (D) guar gum.

Images:

• Fig. 1: Guar split (A) and guar gum powder (B).

**Fig. 2:**
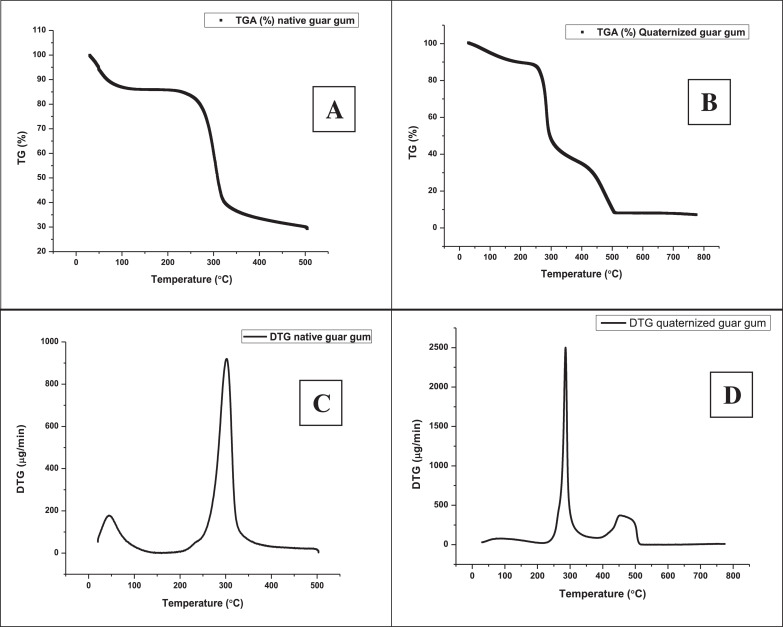
Thermo-gravimetric plots: TGA of native (A) and quaternized (B) guar gum; DTA of native (C) and quaternized (D) guar gum.
• Fig. 3: Activation energy plots of native (A) and quaternized (B) guar gum.

**Fig. 3:**
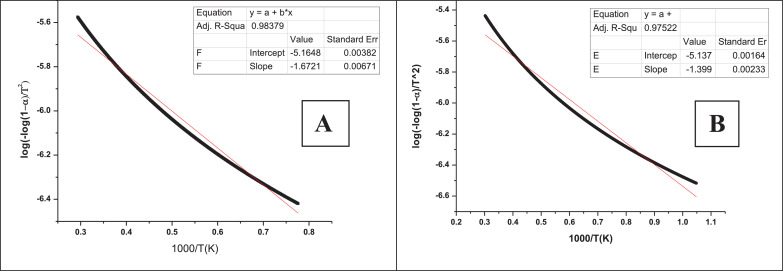
Activation energy plots of native (A) and quaternized (B) guar gum.
• Fig. 4: SEM images of native (A) and quaternised (B) guar gum at different magnification.

**Fig. 4:**
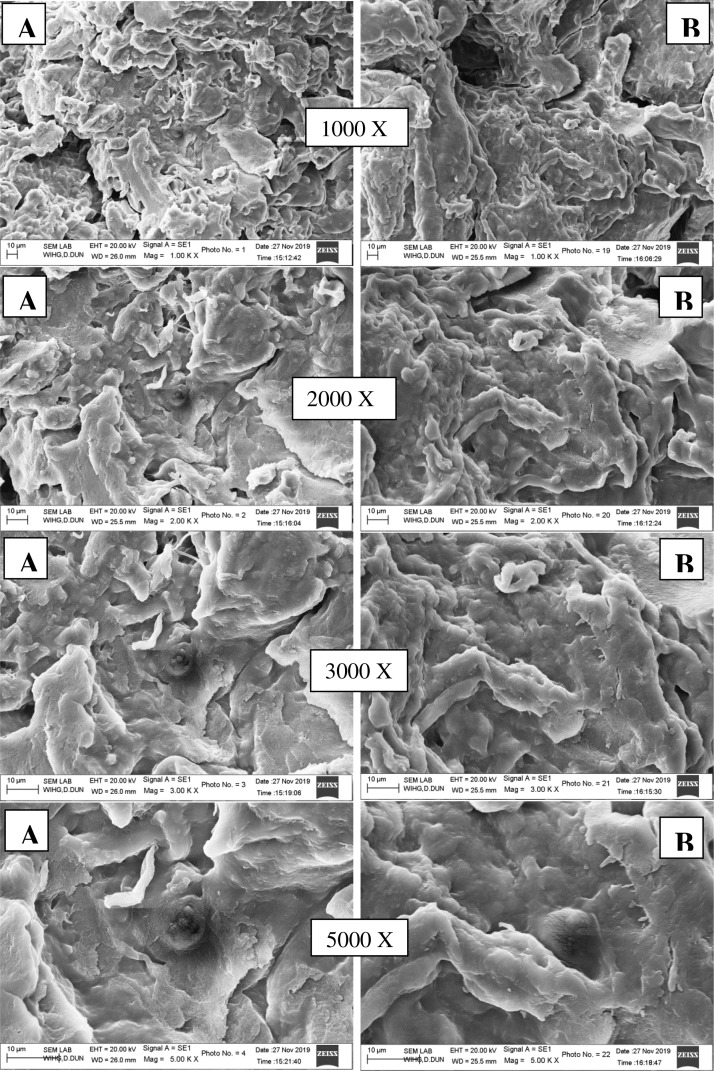
SEM images of native (A) and quaternised (B) guar gum at different magnification.
• Fig. 5: COSY spectrum of native (A) and quaternized (B) guar gum.

**Fig. 5:**
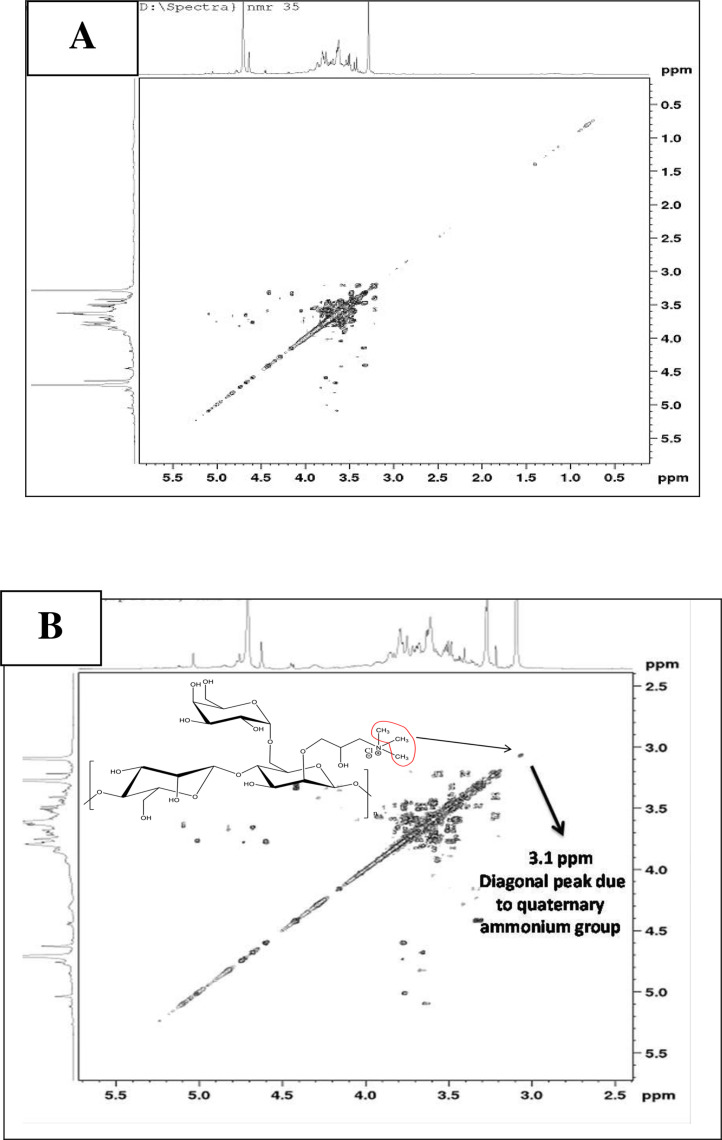
COSY spectrum of native (A) and quaternized (B) guar gum.

Guar splits were ground to obtain guar gum powder. The powdered guar galactomannan was used as starting material for derivatization by introduction of quaternary ammonium groups as described previously [2].

The thermal stability of native and quaternized guar gum was evaluated by TGA analysis of respective samples. Data was acquired in inert atmosphere (argon) using temperature range 35–500 °C. DTA analysis illustrates the temperature at which maximum weight loss occurred. The analysis was performed using conditions similar to TGA acquisition.

Activation energy plots of the native and quaternized guar gum were derived from TGA data of respective samples by adopting the methodology as described in [1].

Surface morphology of native and modified samples were studied by SEM imaging; data was acquired using magnification range of 1000X–5000X.

COSY spectrum of native and modified samples was recorded by hydrolysis of respective polysaccharide samples followed by removal of acid using rotatory evaporator. Acid free samples were subjected to deuterium exchange and spectral recording was done using D_2_O as solvent [2].

Viscosity of 1% (w/v) aqueous solution of native and modified guar gum samples [2] with degree of substitution (DS) ranging from 0.059 to 0.510 was measured by using Brookfield DV-III Ultra Digital Viscometer.

## 2 Experimental design, materials, and methods

Native and quaternized guar gum samples [2] were dissolved in distilled water and precipitated with methanol using the standard protocol. The precipitated polysaccharide was filtered, washed with aqueous methanol followed by pure methanol and dried in an hot air oven. The pure polysaccharides obtained from both native and quaternized guar gum were used for further analyses.

### 2.1 Thermogravimetric analysis

TGA/DTA analysis was carried out using 10 mg of sample in a DTG-60 unit (Shimadzu, Japan) under the argon atmosphere. The scan was carried out at a heating rate of 10 °C/min from 35 to 500 °C. Data obtained in TGA was further analysed to determine activation energy using Coats and Redfern method [1].

### 2.2 Scanning electron microscopy

SEM analysis of native and quaternized guar gum sample was recorded using SEM- Zeiss EVO-40 EP instrument with 10,00,000 X magnification and 30 nm resolution. The powdered native and quaternized samples were coated with gold and images were acquired in the magnification range of 1–5 K.

### 2.3 NMR spectroscopic analysis

The NMR spectra were acquired using a 500 MHz Bruker spectrometer at 25 °C. The native and quaternized products were hydrolysed with 0.5 N HCl. Excess of HCl was removed from hydrolysed mass by repetitive co-distillation with methanol using rotatory evaporator. The acid-free product was subjected to deuterium exchange (three times). The hydrolysed samples of native and quaternized guar (40 mg) were dissolved in 0.75 mL D_2_O (99.95%).

### 2.4 Determination of rheological properties

The viscosity data of 1% aqueous solution (w/v) of native and quaternized products of DS ranging from 0.059 to 0.510 was determined using Brookfield DV-III Ultra Digital Viscometer. Viscosity of samples was recorded at 25±1 °C using spindle 29 at shear rate of 2.5 s^−1^ (Table 1).

**Table 1:** Rheological data of native and quaternized guar gum, Spindle 29, Shear rate 2.5 s^−1^, at 25 °C.

S. No.	Sample code/DS	Viscosity of quaternized guar (Cps)
1	Native guar gum	6721
2	1F1_4_F2_3_F3_2_F4_4_F5_1_/0.059	2105
3	1F1_1_F2_2_F3_2_F4_2_F5_2_/0.370	5632
4	2F1_3_F2_3_F3_1_F4_2_F5_4_/0.510	2741

## Appendix Supplementary materials

Image, application 1
